# Coughing Up a Lung: A Curious Case of Pertussis

**DOI:** 10.7759/cureus.3941

**Published:** 2019-01-23

**Authors:** Mahmoud Y Madi, Manar Y Shahwan, Christine Nayar, Sucharita Kher

**Affiliations:** 1 Internal Medicine, Tufts Medical Center, Boston, USA; 2 Internal Medicine, University of Jordan, Amman, JOR; 3 Internal Medicine, New York University, New York, USA

**Keywords:** pertussis, lung extrusion, whooping cough, cough, liver extrusion

## Abstract

Pertussis is a commonly underdiagnosed infection with incidence that has been steadily rising in adolescents and adults over the last three decades. Some reports suggested cyclical pattern of pertussis infection occurrence with peaks every two to five years. The complications of pertussis can be infectious or mechanical in the setting of persistent cough. We report an unusual case of a 67-year-old woman who presented with combined lung and liver extrusion in the setting of pertussis infection. This article will review the systematic approach of diagnosis and management of pertussis infections in adults.

## Introduction

Pertussis infection can be complicated by a wide spectrum of conditions including pneumonia, otitis media, subconjunctival hemorrhage, abdominal wall hernias, rib fractures, urinary incontinence, and lumbar strain [1]. More vigorous coughing can be associated in more severe cases with intracranial hemorrhage, vertebral or carotid artery dissections, and seizures. Lung or liver extrusion is a very rare mechanical complication related to the classical whooping cough which is the hallmark of pertussis infection. We report a case of a 67-year-old woman with a history of asthma and tobacco smoking presenting with rib fracture leading to lung and liver extrusion in the setting of pertussis infection and persistent cough. We also provide a literature review of the systematic approach to diagnosis and management of pertussis infection with special attention to the adult population.

## Case presentation

**Figure 1:**
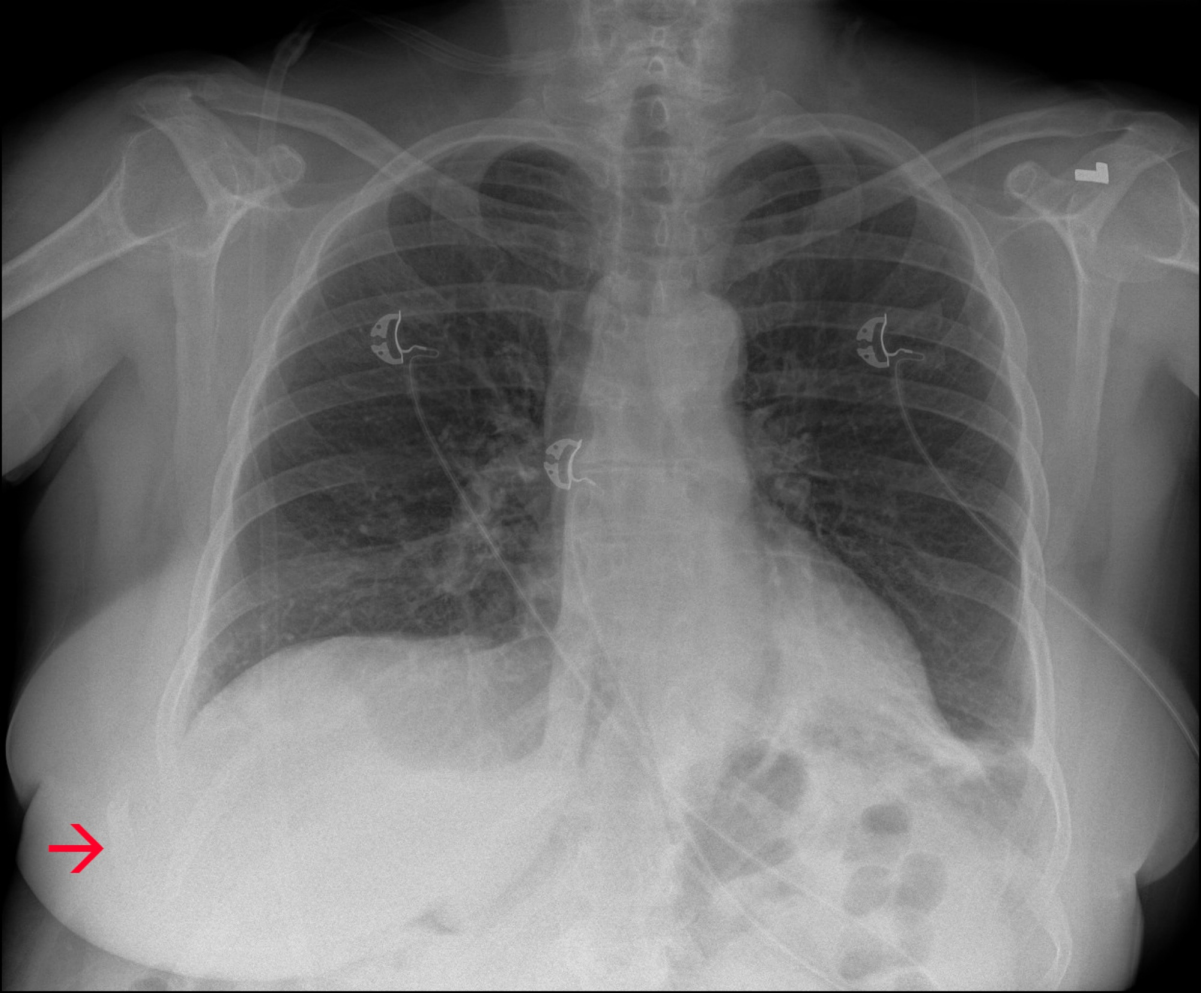
Chest X-ray. Chest X-ray revealing a right-sided ninth rib fracture (red arrow) with lateral displacement of the rib into the surrounding soft tissue. There is no evidence of pulmonary infiltrates.

A 67-year-old woman with a past medical history of asthma, tobacco smoking, hyperlipidemia, and hypertension presented to the hospital with persistent dry cough. Two weeks prior to presentation, she suddenly developed a violent dry cough that progressively worsened. The cough was constant throughout the day and was present on most days. She also described prodromal symptoms of malaise, myalgias, nasal congestion, and runny nose that preceded the cough. No traumas were reported. Five days after symptoms onset, she developed severe right-sided chest pain with extensive bruising on the right side of her chest extending down to her abdomen and right upper thigh. She was evaluated by her primary care physician who obtained a chest X-ray that revealed a right-sided ninth rib fracture (Figure 1) for which she was treated conservatively with acetaminophen and as needed with ibuprofen. She was prescribed a codeine-based cough syrup for her presenting symptom.

**Figure 2:**
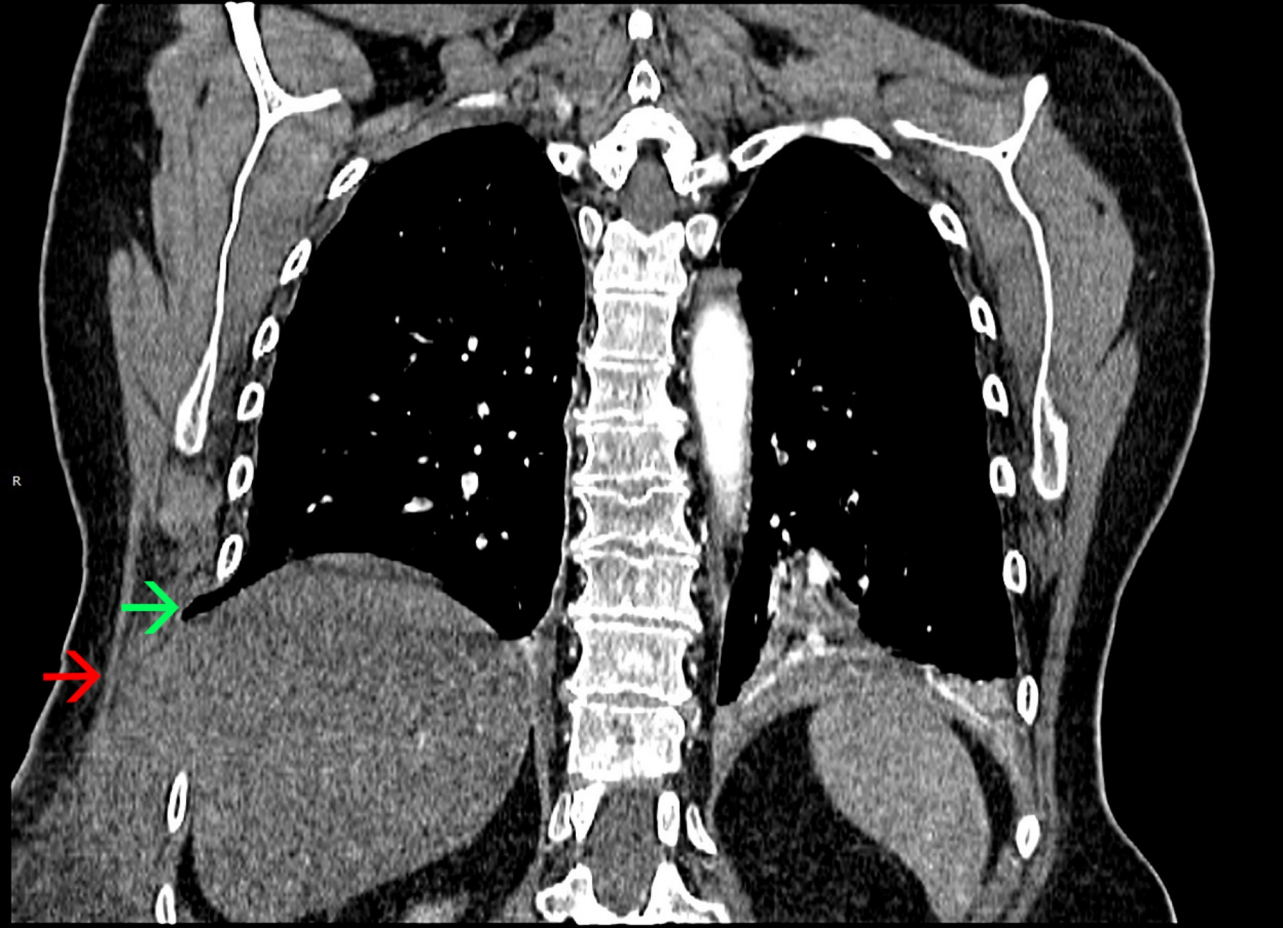
Computed tomography (CT) of the chest. CT scan of the chest revealing a right-sided ninth rib fracture complicated by right lower lung extrusion (green arrow) and liver extrusion (red arrow) between the right eighth and tenth ribs.

Four days later, the patient presented to the emergency department for worsening right-sided chest wall pain and persistent dry cough. Her physical exam revealed normal vital signs, normal lung sounds with good air movement bilaterally, absence of wheezing and significant purple-colored ecchymosis starting at the right-side of the chest at the level of the ninth rib extending down to the right-side of the abdomen and the right upper thigh. Laboratory work-up was non-revealing with a normal complete blood count and chemistry. Computed tomography (CT) scan of the chest, abdomen and pelvis showed an acute fracture of the right ninth rib associated with a muscular abdominal wall hematoma in addition to right lower lung and right liver lobe extrusion between the right eighth and tenth ribs (Figure 2).

Given these findings, a respiratory viral panel swab was obtained for testing, and it was positive for *Bordetella pertussis* using the polymerase chain reaction (PCR) method. Further exploration of the patient's prior vaccination history revealed that she did not receive the tetanus, diphtheria, and acellular pertussis (Tdap) booster vaccine in her adulthood. She was diagnosed with pertussis causing persistent cough, leading to right-sided ninth rib fracture with extrusion of the right lower lung and liver. She was treated with azithromycin for a course of five days in addition to cough suppressants. Her pain was managed with acetaminophen and as needed oxycodone. Her abdominal wall hematoma was managed conservatively with serial monitoring of her hemoglobin levels which remained stable without requiring any blood transfusions. Chemoprophylaxis with azithromycin was provided to the close contacts of the patient. Surgical consultation was obtained, and the decision was to defer the elective surgical repair of extruded viscera until cough resolution.

On follow-up few weeks after discharge, the patient's symptoms dramatically improved. She underwent a successful surgical repair of the visceral extrusions.

## Discussion

Pertussis is a well-studied respiratory illness caused by *Bordetella pertussis*, a bacterium that is strictly pathogenic to humans. There are eight different Bordetella species [1]. Transmission is via aerosolized respiratory droplets with an average incubation period of seven to ten days (range: one to three weeks). The incidence of pertussis steadily increased in the last few years in the USA with 27,550 reported cases in 2010 and more than 41,000 cases in 2012 [2]. The incidence is estimated to be around 53.6 and 3.9 per 100,000 adolescents and adults, respectively. It is more commonly a disease of the younger populations; however, it is likely that pertussis is underdiagnosed in adults confounding the data we have.

It is well known that the immunity acquired from Tdap vaccine wanes with age, lasts five to ten years, and a potential gap exists between the last childhood dose of the vaccine and the suggested booster dose [3]. Age older than 65 years, history of smoking, and pre-existing asthma all predispose to more severe disease when compared to younger and previously healthy adults [4]. A single Tdap booster is recommended between ages 19 and 64.

The clinical course of pertussis infection follows three phases. The catarrhal phase is one to two weeks of nonspecific symptoms including rhinorrhea, nasal congestion, malaise, myalgia, and mild cough. The paroxysmal phase often starts during the second week with paroxysmal whooping expiratory cough which might last up to three months if left untreated. The convalescent phase is when resolution of the cough occurs gradually over one to two weeks [5].

Diagnosing pertussis requires a high clinical level of suspicion. The Center for Disease Control (CDC) clinical case definition is a cough illness lasting two weeks without an apparent cause and with one of the following symptoms: paroxysms of coughing, inspiratory whoop, or post-tussive emesis. PCR and bacterial cultures are the best methods of diagnosing pertussis in the first four weeks with sensitivity being the highest in the first two weeks [6]. Serology is useful when the disease is suspected after four weeks. Most individuals will clear the infection by six weeks; however, antibiotic therapy is proven to shorten the duration and the severity of the illness if given in the earlier phases of disease (generally up to three weeks as onset would benefit the most). Macrolides are the antibiotics of choice for treatment of pertussis infection. It is recommended to use azithromycin 500 mg once a day for five days as a first-line therapy. One double strength tablet of trimethoprim-sulfamethoxazole for 14 days is an acceptable alternative for people with macrolide allergies [7]. Cough suppression is important to avoid mechanical complications. Asymptomatic close contacts should be offered chemoprophylaxis with the same antibiotics used for the treatment of pertussis. Symptomatic close contacts warrant treatment as cases of pertussis.

## Conclusions

It is important to remember pertussis as a potential cause of severe persistent cough especially in the setting of asthma. Potential mechanical complications of the classical whooping cough can include rib fractures and visceral extrusion which might warrant surgical repair. When evaluating a patient with dramatic cough, a systematic approach to diagnosis of pertussis, as described above, is necessary to design an appropriate management plan. This includes having a high suspicion for diagnosing this commonly missed illness, using the clinical definition of a pertussis case to assess for the presence of this disease and confirming it with either bacterial cultures, PCR, or serology depending on the duration of the illness and empirical treatment until diagnosis is confirmed if the clinical suspicion is high enough.
